# Diversity, Concentration and Dynamics of Culturable Fungal Bioaerosols at Doha, Qatar

**DOI:** 10.3390/ijerph18010182

**Published:** 2020-12-29

**Authors:** Raghdaa K. Fayad, Roda F. Al-Thani, Fatima A. Al-Naemi, Mohammed H. Abu-Dieyeh

**Affiliations:** Department of Biological and Environmental Sciences, College of Arts and Sciences, Qatar University, Doha 2713, Qatar; 200551272@student.qu.edu.qa (R.K.F.); ralthani@qu.edu.qa (R.F.A.-T.); Fatima.Alnaemi@ccq.edu.qa (F.A.A.-N.)

**Keywords:** aerobiology, aeromycology, seasonal variations, intra-diurnal variations, CO_2_ concentration, meteorological parameters

## Abstract

This research was conducted to investigate the dynamics of airborne fungi using viable culture collection and in respect to different abiotic variables, including seasonal and intra-diurnal variations. A gravimetric method was used to sample airborne fungal deposition on potato dextrose agar plates on alternate days, for a year between April 2015 to March 2016. From 176 settle plate exposures, a total of 1197 mould and 283 yeast colony-forming units (CFU), 21 genera and 62 species were retrieved. The highest fungal spore count was recorded in February 2016, whereas the lowest count occurred in August 2015. The main constituents of the fungal airspora were attributed to *Cladosporium* (60.2%), *Aspergillus* (10.4%), *Fusarium* (9.4%), *Alternaria* (8.5%), and *Ganoderma* spp. (2.3%). Temperature was negatively correlated with total colony count (*r* = −0.231, *p* ≤ 0.05) or species richness (*r* = −0.267, *p* ≤ 0.001), while wind speed was positively correlated with total colony count (*r* = 0.484, *p* ≤ 0.001) or species richness (*r* = 0.257, *p* ≤ −0.001). The highest dispersal of fungal spores was obtained at 18:00, whereas the lowest fungal spores release was recorded at 00:00 (midnight). There were no significant differences in species composition and richness of the airborne fungal population between two study sites, the Industrial area and Qatar University Campus. The count of *Alternaria* spp. and *Fusarium* spp. were significantly higher at the Industrial area site, which corresponds to a higher CO_2_ level than the Qatar University site. This study lays the foundation for future work to assess the implications of such aeromycological data on public health.

## 1. Introduction

Aeromycology is the scientific study of airborne fungal spores, including their release, concentrations, composition and the parameters affecting their dynamics in the atmosphere [1]. As a branch of aerobiology, aeromycological studies help aerobiologists, plant pathologists and allergist by providing baseline information on the predominance of fungal populations and their dynamics due to abiotic interactions [1].

Airborne fungi cause negative effects on humans’, animals’ and plants’ health [1,2]. The allergen production by fungi is influenced by their life cycle following atmospheric release during spore germination [3]. Fungal spores are small in size and exist in respectable quantity in the atmosphere. They are implicated in respiratory allergies, and symptoms include asthma, allergic rhinitis and bronchitis [4]. It has been demonstrated that environmental factors, such as meteorological and seasonal climate conditions, vegetation, air pollution and human activities influences airborne fungal spore formation and transport [5,6,7]. Several studies were conducted with the aim of detecting the dynamics of aeromycota in associations with the biotic and abiotic factors of their surrounding environment in many parts of the world, some of these include India [8], Australia [9], Chile [10], Poland [11] and China [12]. However, to the best of our knowledge, only few aeromycological studies were conducted in the Middle East compared to other continents, these comprise Israel [13], Jordan [14,15], Egypt [16], Turkey [17], Iran [18], Saudi Arabia [19,20] and Kuwait [21,22]. Although the impact of meteorological factors on air quality is considerable and well established, studies concerning the relationship between fungal counts and air pollutants are still few [23]. The increasing level of atmospheric carbon dioxide (CO_2_) could significantly affect airborne fungal growth and subsequently influence their dispersal rate [24]. As a result of increasing atmospheric CO_2_, which often elevates the carbon-to-nitrogen ratio and plant biomass, the sporulation rate of *Alternaria alternata* was substantially increased [25].

In Qatar, only one study was carried out on the dynamics of airspora of Doha for a whole year (1997–1998) [26]. The author concluded that the mycoflora in the air of Doha exhibited a seasonal and diurnal variation in which *Cladosporium*, *Alternaria* and *Ulocladium* were the most abundant genera in the atmosphere of Doha [26]. Qatar is a peninsula located in the Arabian Gulf region. Plant coverage is infrequent and scattered due to the shortage in water supplies, mainly varied between herbaceous plants, shrubs and a limited number of tree species [27]. Doha is the capital of Qatar and the most popular city, and the increasing anthropogenic activities arising from road and mass housing constructions within the last 15 years could influence species dynamics.

This study aims to: (1) provide baseline knowledge about density, diversity and dynamics of airborne fungal spores in the atmosphere of Doha using settle plate exposures, (2) investigate variations in seasonal and intra-diurnal patterns and to correlate variations with meteorological factors and (3) correlate the variations in species composition and abundance of airborne fungi with atmospheric CO_2_ concentration.

## 2. Materials and Methods

### 2.1. Study Area

Qatar is a peninsula occupying an area of more than 11,000 km^2^ and a coastline of 900 km in length. It lies in the Arabian Peninsula at 25°35′48″ N and 51°18′39″ E and is connected to Saudi Arabia in the south, bordered by a very shallow, semi-enclosed sea characterized by hyper-salinity (39–41) practical salinity unit (psu) for surface water) [28]. Geographically, it is a flat, rocky and arid desert and in the south, sand dunes are the predominant features. As a subtropical desert, Qatar is hot and has dry weather, the annual rainfall is about 81 mm, and the average maximum air temperature is 31 °C, although the maximum temperature could exceed 47 °C [29]. Doha is the capital of the state, located on the eastern Qatari coastal line at 25°17′12″ N, 51°32′0″ E. It is the most urbanized and populous city in the state, the population in the country until February 2015 was 2,116,400 [30].

The present study was carried out at Qatar University, which is located on the northern side of the capital Doha at 25°37′47″ N, 51°49′03″ E. The main habitat and associated vegetation in the northern part of Qatar is characterized by ‘rodat’ areas, in which compact soils are common. This type of soils usually supports more moisture and organic matter than other desert parts. Vegetation type ranges from trees and shrubs to grasses and herbs, and common species include *Acacia* spp., *Prosopis juliflora*, *Ziziphus nummularia* and *Lycium shawii* [31]. Because this area accommodates larger rodats, they have been utilized as farms for growing crops [28]. Within the campus of Qatar University, naturally occurring plants are widespread; additionally, many species associated with man-made and man-influenced sites, such as gardens, green houses and roadsides, are very common.

### 2.2. Seasonal Variations in Airborne Fungal Populations in Doha City

A gravimetric method (settle plate exposure) using a Petri dish was carried out directly on the roof of the building of Qatar University (about 12 m above ground level, including the height of the metal mount). One Petri dish (9 cm diameter) containing Potato Dextrose Agar (PDA) (Hi-Media Laboratories, Mumbai, India) amended with chloramphenicol (1 g/L), was exposed for 15 min, which resulted in more accurate colony counts, at 15:00 on each of 3 days per week (alternate days), from the beginning of April 2015 to the end of March 2016. Total exposures of 176 plates were collected during the year of study. After sampling, the plates were incubated at 25 °C for 3–5 days.

Daily meteorological data from Qatar University weather station were supplied by the Weather Record Department, Doha, Qatar. The meteorological data considered are minimum and maximum daily temperature, minimum and maximum relative humidity, daily rainfall, wind direction and wind speed.

### 2.3. Intra-Diurnal Variations in Airborne Fungal Populations in Doha City

A period of two months, February and March (2016), was chosen to investigate intra-diurnal variations in fungal populations of Doha, mainly because this period was preliminarily explored as a period of greatest diversity in species composition in Doha’s atmosphere. One Petri dish (9 cm diameter) containing PDA was exposed to air for 15 min at 6-h intervals (at 06:00, 12:00, 18:00 and 00.00) during the period of 1 February to 31 March 2016. After sampling, the plates were incubated at 25 °C for 3–5 days. During the whole period, a data logger (OMEGA Engineering INC., Swedesboro, NJ, USA) was installed at the site of collection to monitor temperature and relative humidity at intervals of one hour.

### 2.4. Effect of CO_2_ Concentration on Species Composition and Abundance of Fungal Spore Populations

Two locations at Doha city were chosen to investigate the impact of CO_2_ level on species composition and abundance of fungal spore populations. Qatar University is one of these locations, which is relatively far from industrial areas (~25 km), and the other is close to the central industrial area (Industrial Area). Industrial area is a district of Doha, Qatar, with coordinates 25°10′3″ N and 51°26′22″ E.

During the period of 1 February to 31 March 2016, Petri dishes (9 cm diameter) containing PDA were exposed to the air for 15 min on alternate days. In respect to the two locations and during sampling, the plates were opened at the same time and for the same exposure period (15 min). After sampling, the plates were brought to the lab using an icebox and then incubated at 25 °C for 3–5 days. The grown fungal colonies for each species were counted and the total colony counts were recorded. A portable CO_2_ data logger (CO2METER Inc., Ormond Beach, FL, USA) was used to record CO_2_ concentration every minute during the collection period. The average of the 15 readings per sampling was recorded.

### 2.5. Identification of Airborne Fungal Spores

The fungal grown colonies for each species were counted and recorded. For classification, fungal colonies were isolated and purified on Potato Dextrose Agar (PDA) and other selective agars like Malt Extract Agar (MEA) (HiMedia Laboratories Pvt.Ltd, Maharashtra, India) and Czapek’s/Rose-Bengal agar (HiMedia Laboratories Pvt.Ltd, Maharashtra, India), then, they were incubated for 3–5 days at 25 °C. Identification was based on the macro- and micro-scopic features following the keys and description given by many authors [32,33,34,35,36,37,38,39].

### 2.6. Statistical and Data Analysis

Statistical analyses were performed to correlate the mean daily fungal spore counts and species composition with the daily data of the meteorological parameter of the same day and for the whole year using Pearson’s correlation coefficient. Similar correlation analyses were performed to investigate the effect of CO_2_ on species composition and abundance. To compare the CO_2_ concentration in the two study sites and as normality failed, a Mann–Whitney test on rank was applied based on 22 sampling exposures from each site. Analysis of variance (ANOVA) was performed to study the significant effect of intra-diurnal periods on deposition densities and species richness. The Shapiro-Wilk test was used to test the normality of data and Tukey’s test was used to separate the means at *p* ≤ 0.05. Temperature and relative humidity data from the data logger were averaged for each sampling time (e.g., between 18:00 and 24:00) and Pearson’s correlation coefficient was performed to study the correlation between colony count of each sampling exposure with the average temperature or relative humidity. The Jaccard similarity coefficient was applied to compare the similarities in species composition (including species identified at the specific level and other taxa) among the four diurnal periods, as follows: Jaccard similarity coefficient = (c/(a + b) − c) × 100, c = number of common species between any two time periods (a and b). A T-test at significance level *p* = 0.05 was accomplished to study the significance in daily colony counts between the two study sites according to differences in CO_2_ concentrations.

## 3. Results

### 3.1. Seasonal Variations in Airborne Fungal Populations in Doha City

From the air of Doha, the total colony count number retrieved from 176 exposure samplings during the year of study (1 April 2015 to 31 March 2016) was 283 yeast and 1197 mould colony-forming units (CFU). The mould colonies belong to 21 genera and 62 species (Table 1).

**Figure 1:**
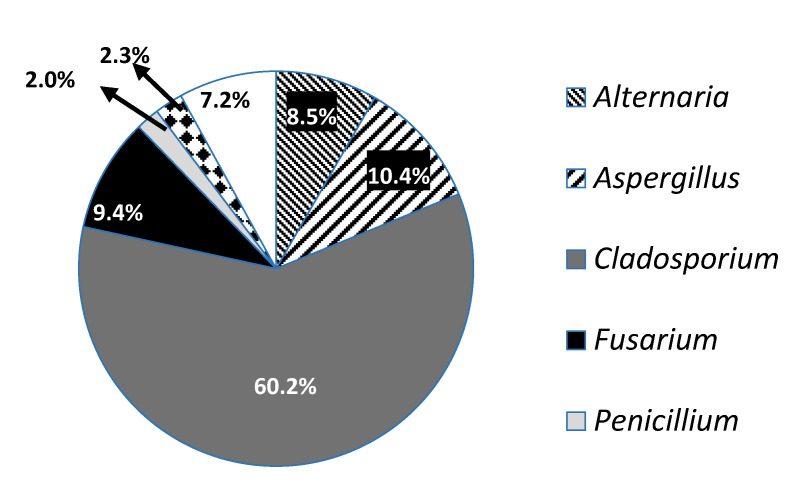
The composition of fungal constituents (%) in the atmosphere of Doha City during 1 April 2015 to 31 March 2016, using the gravimetric method (a total of 176 exposure samplings).

The maximum count of airborne fungal spores was recorded in February 2016, whereas the minimum occurred in September 2015. The main constituents of the airborne fungi population in the atmosphere of Doha were attributed to the genera: *Cladosporium* (60.2%), *Aspergillus* (10.4%) *Fusarium* (9.4%), *Alternaria* (8.5%), *Ganoderma* spp. (2.3%) and *Penicillium* (2.0%) (Figure 1).

**Figure 2:**
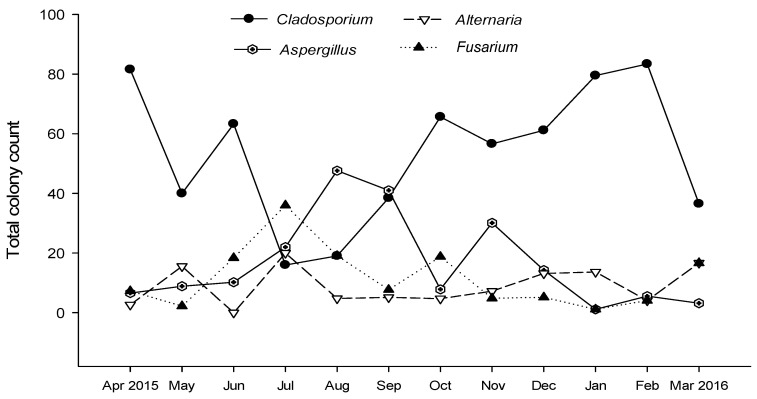
Seasonal variation of the four most common fungal genera in the atmosphere of Doha during the year of study (1 April 2015 to 31 March 2016) using the gravimetric method (a total of 15 exposure samplings per month).

*Cladosporium* was the most common fungal taxa in the air of Doha, comprising around two thirds of the total colony counts representing five species. *C. cladosporioides* was the most dominant species, with 70.5% of the total of the genus *Cladosporium*, followed by *C. macrocarpum* with 9.2% and *C. sphaerosprmum* with 6.4%. *Cladosporium* had double peaks of occurrence (April and February) and the lowest occurrence in July (Table 1; Figure 2).

*Aspergillus* sp. was the second predominant fungal genera represented by ten species (Table 1). *Aspergillus* achieved the highest deposition density in August 2015 and the lowest in January 2016 (Figure 2) *A. flavus* abundance was 46.5% of the total among the genus *Aspergillus*, then *A. sydowii* with 25.2% (Table 1; Figure 1). *Fusarium* was ranked as the third among the most common airborne fungi in Doha. Most recovered colonies of *Fusarium* spp. were identified at the genus level, however *F. oxysporum* and *F. chlamydosporum* were the most abundant and frequently recorded (Table 1). *Alternaria* represented the fourth most prevalent fungal taxa, with seven identified species. The spore count of *A. alternata* put up with more than half of the whole genus (61.4%), followed by *A. chlamydospora* (12.8%), then *A. infectoria* (11.9%) (Table 1; Figure 1). Both *Alternaria* and *Fusarium* reached their maximum deposition densities in July 2015, the spore count of *Alternaria* spp. declined in June 2015, while *Fusarium* colony counts diminished in January 2016 (Figure 2). Interestingly, *Ganoderma* spp. was ranked fifth among the most predominant fungi in the atmosphere of Doha, with 2.4% abundance, even though they exclusively appeared in February and March 2016 of the whole year (Table 1; Figure 1).

### 3.2. Weather Interactions with Airborne Fungi of Doha

According to the correlation analyses between weather parameters and the incidence of the main fungal taxa, total daily or monthly colony count and species diversity are presented in Table 2. Both daily and monthly counts of either of *Cladosporium* or *Alternaria* spores showed significant negative correlations (*p* ≤ 0.05) with daily maximum, minimum and mean temperatures. While the correlations of *Cladosporium* colony count with both daily or monthly relative humidity and rainfall were not significant, the monthly rainfall data showed a highly significant correlation (*p* ≤ 0.01) with *Alternaria* colony counts. A high significant (*p* ≤ 0.01) positive correlation occurred between daily counts of *Cladosporium* and *Alternaria* and wind speed (Table 2). On the contrary, *Aspergillus* deposition density showed non-significant correlations with any of the studied weather parameters (Table 2)*. Fusarium* daily colony counts had the only significant correlation (*p* ≤ 0.01) with the daily wind speed data (Table 2). On the other hand, total daily colony counts of all encountered fungi showed significant negative correlations (*p* ≤ 0.05) with all daily temperature parameters. A high significant positive correlation (*p* ≤ 0.01) was also observed between the total daily or monthly colony count and daily or monthly wind velocity (Table 2), and a non-significant correlation with either of daily mean relative humidity or rainfall. However, no significant correlation occurred between total colony counts and either relative humidity or rainfall. Daily temperature parameters showed negative correlations with fungal species diversity of Doha’s atmosphere, while daily wind speed data exhibited a significant positive correlation (*p* ≤ 0.01) with species diversity (Table 2). There was no significant correlation between air-borne fungi and wind direction (Table 2).

### 3.3. Diurnal Variations of Fungal Spore Populations in the Atmosphere of Doha

During the two months of study (1 February to 31 March 2016), the mean daily maximum temperature ranged from 24.6 to 27.3 °C, the mean daily minimum temperature ranged between 15.0 and 18.7 °C. The daily relative humidity varied from 39% to 84%. Significant differences (*p* ≤ 0.05) in colony count and species diversity were found among the four studied periods. The highest colony count and species diversity were recovered at 18:00, while the lowest were reported at 00:00 (midnight). No significant differences in colony counts as well as species diversity between 12:00 and 18:00 were observed (Table 3). The highest similarity coefficient in species composition was obtained between 00:00 and 06:00, while the lowest was detected between 18:00 and 00:00 (Table 4).

**Figure 3:**
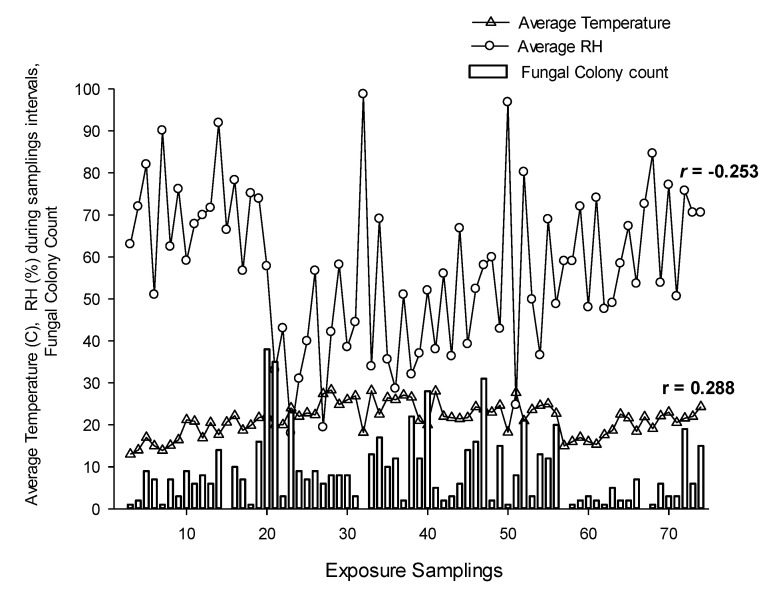
Variations in fungal colony counts in each exposure sampling with respect to changes in average temperature and relative humidity (RH) during the 6 h intervals. Correlation coefficient values (*r*) are significantly different between mean daily counts and mean daily temperature and mean daily relative humidity at *p* ≤ 0.05, according to Pearson’s correlation coefficient.

**Figure 4:**
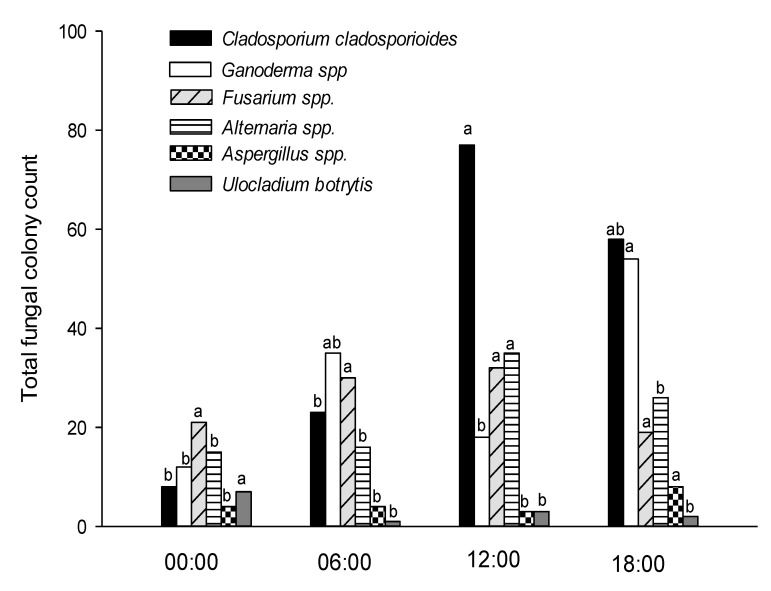
Diurnal variations in total fungal colony counts of the most common species in the atmosphere of Doha during the period 1 February to 31 March 2016. A total of 72 exposure samplings. Within each fungal taxa, values with common letters are not significantly different at *p* ≤ 0.05, according to Tukey’s test. Significant correlations were reported between mean daily temperature and the mean daily counts of *Alternaria* spp. (*r* = 0.316) and mean daily counts of *Ganoderma* spp. (*r* = 0.237) at *p* ≤ 0.05, according to Pearson’s correlation coefficient.

The mean daily colony count was negatively correlated (*p* ≤ 0.05) with mean daily relative humidity and positively correlated (*p* ≤ 0.05) with mean daily temperature (Figure 3). However, no significant correlations were found between species diversity and any of the weather parameters. Interestingly, the mean daily counts of the genera *Alternaria* and *Ganoderma* have shown significant positive correlations (*p* ≤ 0.05) with the mean daily temperature. The most abundant fungal taxa retrieved throughout the two months at the four periods were *Cladosporium cladosporioides, Alternaria* spp., *Fusarium* spp., *Ganoderma, Ulocladium botrytis* and *Aspergillus* (Figure 4).

*Cladosporium cladosporioides* represented a mid-day (12:00) peak and had a minor peak at 18:00, while its deposition density significantly declined (*p* ≤ 0.05) at midnight (00:00) and in the early morning hours (06:00). *Ganoderma* spp. significantly peaked (*p* ≤ 0.05) at 18:00 and showed similar abundance among the other three periods. *Alternaria* had a significant mid-day (12:00) peak pattern, while the colony count significantly declined in the other three periods. No significant differences in *Fusarium* colony count were reported among the four studied periods, while *Aspergillus* had a major peak pattern at 18:00 and *Ulocladium botrytis* at midnight (00:00) (Figure 4).

### 3.4. The Impact of Atmospheric CO_2_ Concentration on the Dynamics of Airborne Fungi

**Figure 5:**
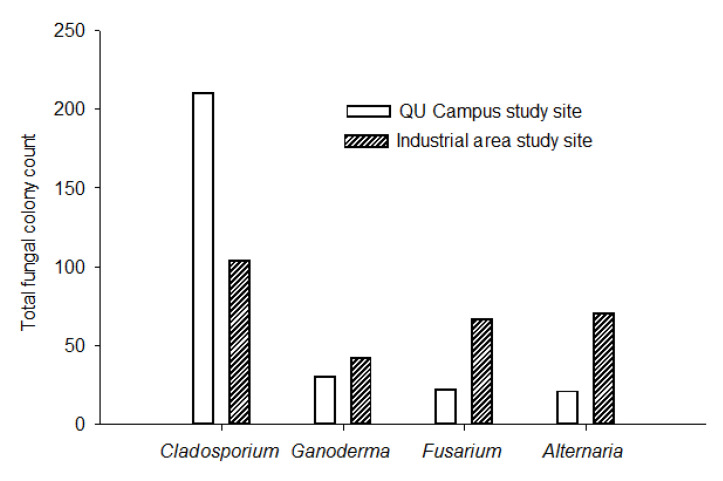
Fungal taxa that showed significant abundance differences between the two study sites (according to the t-test of daily colony count at *p* = 0.05) during the period of 1 February to 31 March 2016, with a total of 22 exposure samplings.

The two-month study (1 February to 31 March 2016) revealed that 12 fungal genera and 31 fungal species were collected from the Qatar University Campus site, while 16 fungi genera and 35 species were collected from the Industrial Area site. No significant differences were observed in the total number of colonies and fungal species between the two study sites, although daily concentration of CO_2_ was higher (according to average, median and range values of CO_2_) at the Industrial area site (average = 513 ± 167 ppm) than Qatar University Campus (average = 335 ± 92). Applying the non-parametric Mann–Whitney test on rank indicated a significant difference (*p* < 0.001, *n* = 22) in CO_2_ concentrations between the two sites. The common and most abundant fungal taxa recorded in the two study sites were *Cladosporium, Ganoderma, Fusarium* and *Alternaria.* Corresponding to CO_2_ concentrations, the daily colony count of *Cladosporium* was significantly higher at Qatar University Campus than the Industrial area (*p* ≤ 0.05) (Figure 5). Contrary to *Cladosporium,* the deposition density of *Fusarium* and *Alternaria* were significantly higher (*p* ≤ 0.05) at the Industrial area site (Figure 5).

**Figure 6:**
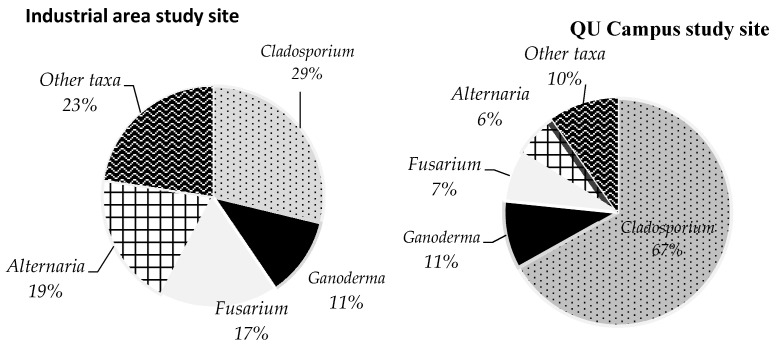
Pie graph to show the main constituents of fungal genera and their relative abundances in the atmosphere of the two study sites during the period of 1 February to 31 March 2016, with a total of 22 exposure samplings.

The main constituents of fungal genera and their abundance rate in the atmosphere of the two study sites were widely variable between the two sites (Figure 6). *Cladosporium* was prominently higher in relative abundance (67%) at the University campus, constituting about two thirds of fungal composition. Other fungi like *Ganoderma*, *Fusarium* and *Alternaria* were also part of the main composition, with relative abundances of 11%, 7% and 6%, respectively. Other taxa at the University Campus had 10% abundance (Figure 6), while *Cladosporium* remain the main constituent of fungal taxa at the Industrial Area site, however, relative abundance (29%) was very low compared to the University site (67%) (Figure 6). Interestingly, *Ganoderma* showed similar relative abundances (11%) in the two sites (Figure 6). The fungal genera, *Alternaria* and *Fusarium*, were considered to be the main constituents with relative abundances of 19% and 17%, respectively; however, other taxa are still represented by 23% (Figure 6).

## 4. Discussion

### 4.1. Seasonal Dynamics of Air-Borne Fungi of Doha

During the year of study, a total of 1197 colony counts and 21 genera assigned to 62 species were retrieved. *Cladosporium*, *Aspergillus Fusarium*, *Alternaria*, *Ganoderma* spp. and *Penicillium* were the main components of the air mycoflora of the Doha area. Several studies demonstrated the presence of the above-mentioned fungal taxa as part of the main aeromycota composition in different Middle Eastern countries [15,19]. According to another study conducted in Doha, Qatar [26], the major airborne fungal genera obtained were *Cladosporium*, *Alternaria* and *Ulocladium*. Although the method of collection used in Al-Subai’s study is similar to the one used in the present study, there exist some differences in the experimental setup and design. For instance, the collection height used by Al-Subai was 1.5 m, but in the present study, a height of 12 m above the ground level was used, which is considered more realistic in such aerobiological studies [10]. Another major difference is the culture media used, 1% glucose-Czapek’s agar + 0.05% yeast extract culture medium versus Potato Dextrose Agar (PDA), in this study. Both, the height level of collection and culture media used are considered variables that affect the sampled species composition and abundance of air mycoflora [40,41,42,43,44]. These, in addition to the increased anthropogenic activities over the last 17 years that occurred in Qatar, may explain the differences in the results obtained.

Our study showed that *Cladosporium* was the most common fungal genus and commonly occurred throughout the study periods with double peaks, one in April and another in February. Many authors presented *Cladosporium* as the main air mycoflora in different countries [14,26,45]. Many factors might support the abundance of *Cladosporium* throughout the year. It has been demonstrated that the structural features of *Cladosporium* conidia, such as a chlamydospore-like structure, make them more resistant to solar radiation and physicochemical agents [26,46]; however, other authors explained this phenomenon by referring to the small size, thin exine and smooth wall of the *Cladosporium* spores, which helps in the dissemination of conidia [47,48].

*Aspergillus* sp. was the second predominant fungal genera, with ten species recorded. *Aspergillus* presented a seasonal pattern: the highest deposition density was in August and the lowest in January. *Aspergillus* sp. was the predominant taxa in many countries’ outdoor environments, like Kuwait [22] and Saudi Arabia [19,49].

The abundance of *Aspergillus* sp. in the atmosphere of Doha and other Gulf regions is consistent with the findings of Christensen and Tuthill, who found that the tropical/subtropical habitats accommodated higher numbers of *Aspergillus* species in comparison to temperate regions [50]. Accordingly, they concluded that *Aspergillus* genus is highly abundant in the tropics/subtropics, especially in the saline and cultivated soils.

Additionally, *Fusarium* was ranked as the third airborne fungi. *Fusarium* is a cosmopolitan fungus, which is a well-known plant/animal pathogen [51]. *Fusariun* hardly germinates in low water activity environments [52]. The dispersion of *Fusarium* macrocnidia was found to follow a rain or irrigation event [53]. Indeed, it was reported that *Fusarium* was abundant in the atmosphere of the Zarqa area, Jordan, and this abundance correlated with the continuous drip irrigation of olive plantations in the study site, and the existence of many farms [15]. This may explain the abundance of *Fusarium* in the atmosphere of Doha, since Qatar University campus receives extensive and frequent irrigations to accommodate the need of the vegetation cover (turfgrass areas and others) and the high evaporation rate. However, rainfall might interact with density of *Fusarium*, but it is difficult to achieve any significant correlation trends since the rainfall in Qatar is very low and limited to a few days of the year.

*Alternaria* represented the fourth most prevalent fungal taxa in the air of Doha, with seven species identified. *Alternaria* represented a seasonal trend, with high colony count in May and July, while the colony count declined a little and maintained all over the year with almost minor fluctuations. Similar to *Cladosporium* spores, the conidia of *Alternaria* are of dry spore’s type and dark colour, which tolerate the solar radiation and survive the whole year [26]. Other studies showed that the highest spore deposition of *Alternaria* spp. was reported in July and August, following wet weather [54,55].

*Ganoderma* spp. was one of the major constituents of air mycoflora of Doha (2.4%), but it was exclusively retrieved in February and March. In the present study, *Ganoderma* spp. has been identified for the first time in the atmosphere of Doha. A similar study in Jizan, Saudi Arabia, also found a considerable concentration of *Ganoderma* basidiospores within the airspora [56]. According to the authors, airborne *Ganoderma* spp. was not frequently detected in many countries with similar ecological features in the Gulf region. *Ganoderma* was first suggested to be associated with respiratory allergy in 1952 by Gregory and Hirst [57]. In Jizan, Saudi Arabia, the concentration of *Ganoderma* basidiospores was significantly correlated with the high incidence of asthma in Jizan [56]. The seasonal pattern of *Ganoderma* widely varies worldwide. For example, in Jizan region of Saudi Arabia, the seasonal trend of *Ganoderma* is from October to March, with a peak in December and January when the temperature is moderate and the humidity is relatively high [56]. In Poland, it was found that August is the month of the highest spore incidence, when the weather is warm and humid [11]. In the USA, the spores of *Ganoderma* were reported on more than 95% of the days, of June through October, and reaching a maximum concentration in late August until mid-October, with a positive correlation with temperature and precipitation [58]. In Delhi, India, *Ganoderma* had a seasonal pattern from July to September [59]. The presence of *Ganoderma* basidiospores is determined by the abundance of vegetation coverage [60], and the maximum spore concentration was reported to occur during the rainy months [61]. From the above studies, the incidence of *Ganoderma* basidiospores seems to be more correlated with warm and high moisture or rainfall. Together, these studies corroborate our findings as recorded in February (10%) and March (90%) respectively (Table 2), when the temperature is moderate and the rainfall incidence is the highest in Doha, Qatar.

### 4.2. Fungal Species Fluctuation versus Climatic Factors

According to our results, February was the month of greatest colony count and fungal taxa recorded (Data not shown) in the atmosphere of Doha, and this could be linked to the relatively moderate temperature and high rainfall (Table 2). August and September were the months of lowest fungal abundance and diversity (Data not shown), these months are characterized by relatively high temperature and humidity (Table 2), which may interact to stop the dissemination of fungal spores from their sources (soil and plants) and/or destroy the spores, especially with the accompanying increase in sun radiation during these months.

Equally, an important, significant (*p* ≤ 0.01) positive correlation was obtained between the total daily fungal counts and fungal taxa recorded, which might indicate that similar interactions of weather conditions may determine the dynamics and fluctuation of both parameters. Both daily and monthly counts of either *Cladosporium* or *Alternaria* spores showed significant negative correlations (*p* ≤ 0.05) with daily maximum, minimum and mean temperatures. While the correlations of *Cladosporium* colony count with daily or monthly relative humidity and rainfall were not significant, the monthly rainfall data showed high significant correlation (*p* ≤ 0.01) with *Alternaria* colony counts. A high significant (*p* ≤ 0.01) positive correlation was also observed between daily counts of *Cladosporium* or *Alternaria* and daily wind speed data. Contrary to our results, in Poland, Grinn-Gofroń and Strzelczak found that the concentration of air spores of *Cladosporium* and *Alternaria* were significantly and positively correlated to temperature, but negatively to relative humidity, however the authors could not clearly rank which weather parameter is more important for the viability of those fungal spores in the air [62]. Similarly, in Spain, Sabariego et al., in 2012, concluded that the mean daily count of *Alternaria* was significantly and positively correlated with temperature but negatively correlated to relative humidity, rainfall and wind speed [63]. In Turkey, both *Alternaria* and *Cladosporium* were significantly and positively correlated with temperature and humidity [64]. Our results showed similar trends with other studies that have been conducted in countries with a similar environment to Qatar [18,65,66,67,68]. Hasnain et al., in 2012, demonstrated that the occurrence of *Alternaria* spores is negatively correlated to temperature [65] and Hameed et al., in 2007, mentioned a negative correlation with relative humidity [66]. Studies have found that the concentration of *Cladosporium* spores increased significantly as the temperature decreased, which is in support of our findings [18]. The cumulative rainfall was positively correlated with the level of *Alternaria* incidence [54] and dispersal of *Alternaria* spores was higher in August following a period of wet weather [54]. In general, raindrops accelerate the release of dry spores like *Alternaria* spores. Combined together, these studies are in agreement with our results, suggesting the significant positive correlation between monthly data of *Alternaria* and rainfall.

Interestingly, the meteorological parameters could not influence the spore incidence of *Aspergillus* in the atmosphere of Doha. The nature of *Aspergillus* spores is dry and relatively small, which makes them easily and passively released by even minor wind speed or vibration [7]. Nevertheless, it appears that the inconsistency of the meteorological impacts on the dynamic of these airborne fungal spores may be the result of different climatic conditions in many countries. Our findings agreed with Oliveira et al., who found that *Aspergillus* had no correlation with any of the meteorological parameters [67]. However, contradicting our results, *Aspergillus* level was found to be positively and significantly correlated to temperature, but a negative correlation was observed when compared to relative humidity [40]. In a similar study, *Aspergillus* was said to be positively and significantly correlated to temperature, but negatively to relative humidity [68].

In the present study, the only significant correlation observed in *Fusarium* was to wind speed. Fernando et al., in 2000, noted that similar environmental conditions are favourable to some *Fusarium* species in release of their spores, and although rain could influence sporulation rate in *Fusarium*, it was, however, difficult to address the impact of other factors, such as soil surface temperature and wind velocity [53]. *Fusariun* germination is known to develop well in the presence of high moisture, and, as mentioned earlier, the abundance of *Fusarium* in the study site was attributed to extensive and frequent irrigation activities, aimed at accommodating the need for vegetation cover in addition to high evaporation rate [52].

Total daily colony count of all encountered fungi had negative correlations (*p* ≤ 0.05) with mean daily temperature. A highly significant positive correlation (*p* ≤ 0.01) was obtained between the total daily or monthly colony count and daily or monthly wind speed, and a negative but insignificant (*p* ≤ 0.05) correlation to the daily mean relative humidity and rainfall. However, no significant correlations were reported between total colony count and either relative humidity or rainfall. In agreement with our results, it has been found that the several fungal spore counts are negatively correlated with temperature, though a positive and insignificant correlation was detected with relative humidity [65]. Similarly, Nourian et al., detected a negative correlation between temperature and colony count but positive to relative humidity [18]. Other studies are, however, conflicting with our findings, indicating that concentration of airborne fungal spores was positively and significantly correlated with temperature [68].

Based on our statistical correlation analysis between weather data and fungal airspora, the temperature might have the greatest influence on the dynamics of airborne fungal spores, a fact that has been highlighted by many authors, see References [11,69]. Interestingly, the majority of retrieved fungal airspora are mesophilic (optimal temperature for growth 20–40 °C). Our results showed that relative humidity had no significant impact on the airborne fungi of Doha, which might be attributed to the adaptation of germination, growth and propagation of airborne fungi to relative humidity [18]. Another reason could be that the high temperature plus the sun radiation (not studied in the present study) may be more limiting factors than the humidity in certain months of the year, particularly from July to September. Wind speed was found to significantly exaggerate the number of fungal spores in the atmosphere, which correspond to our findings on all fungal taxa [17,26,63,64]. However, the impact of wind speed was related to the daily data, which means that the impact of wind velocity on the fungal airspora is temporal and, in part, on a daily basis, rather than accumulative effects. On the other hand, wind can act as a spore dispersal and diluting factor at high wind, and hence could significantly reduce spore concentrations [41]. According to Lin and Li, at a wind speed of less than 5 m/s, fungal spores’ concentration decreases, but increases at wind velocity higher than 5 m/s [7]. The change in wind direction had no effect on fungal abundance or diversity. In 1980, McDonald and O’Driscoll found a remarkable impact of wind direction on the count of airborne fungi [70]. On the other hand, and coinciding with our findings, Al-Subai, could not detect any regular correlation between wind direction and the concentration of fungal spores, which might be related to the fact that Doha is a coastal city surrounded by the Arabian Gulf [26]. One of the limitations of this study is the alternate days of sampling, which had interruptions of fungal sampling data, and this might have an effect on the correlation analysis of non-consistent climatic parameters like wind speed and rainfall.

### 4.3. Diurnal Variation of Fungal Spore Populations in the Atmosphere of Doha

In the present study, the atmospheric deposition densities of fungal spores under the influence of intra-diurnal fluctuations were studied during the study period of 1 February to 31 March 2016. Several aeromycological studies have investigated the diurnal periodicity of airborne fungal spores [15,16,26,71]. Our findings revealed an intra-diurnal periodicity pattern of fungal spore deposition densities, suggesting significant differences in the total colony counts and fungal diversity at varying time periods. Both parameters, total colony count and species fungal diversity, peaked significantly in the atmosphere of Doha at 18:00 and declined significantly at 00:00. A negative correlation between colony count and relative humidity was observed, while a positive correlation with temperature was noted. During February and March, the daily temperatures in Doha are less fluctuated and moderate (20–25 °C), which suggests less dryness owing to high temperature and low humidity, which are necessary for spores’ release into the atmosphere. Interestingly, a deposition density gradient of fungal spore was observed, which occurred in upward flux (started from 00:00) coordinating positively with recorded temperature and negatively with relative humidity. Previous studies revealed that the highest concentrations were recorded around 12 h, concomitant with high temperature and decreased relative humidity [26,62,71]. On the other hand, Fengxiang et al. found that the viability of airborne fungi was higher at night than daytime, due to the darkness and high relative humidity, which increase the concentration of fungal spores [72]. However, the period of study is important in determining the diurnal periodicity of fungal spores [14]. Our study was conducted between February and March, which corresponds to the spring period in Qatar, and yet, the diurnal range of minimum humidity could not negatively affect fungal spore release or dissemination, which may allow temperature to be the effective variable in the diurnal cycle of fungal spore dissemination. The highest similarity coefficient was obtained between 00:00 (midnight) and 06:00 (early morning), while the lowest similarity coefficient was detected between 18:00 (afternoon) and 12:00 (noon). The most abundant fungal taxa were *Cladosporium cladosporioides, Alternaria spp., Fusarium spp., Ganoderma, Ulocladium botrytis* and *Aspergillus*. *Cladosporium* and *Alternaria* were found in higher deposition densities at mid-day. These two fungal taxa are of clinical importance due to their allergenic properties [73]. Elsewhere, different studies found that the maximum concentrations of *Cladosporium* and *Alternaria* also occur at mid-day [74].

Consistent with our findings, *Cladosporium* and *Alternaria* were found to have a positive correlation with temperature but were negatively correlated with relative humidity [75,76]. For *Cladosporium,* spores are dry air, thus, mid-day weather conditions, when the temperature is relatively high and relative humidity is low, present the optimal dispersal condition. For *Alternaria,* Recio et al. reported correlation coefficients in terms of maximum, minimum and mean temperature, and accordingly, they suggested that the concentration of *Alternaria* spore is a function of temperature [75]. It was demonstrated that the maximum release of *Ganoderma* spores occurred when the humidity increased and temperature diminished [76]. Similarly, Calderon et al. concluded that the release of basidiospores are negatively correlated to temperature and declines at higher temperatures of more than 27 °C [77]. This is in agreement with our findings: *Ganoderma* was only detected in February and March with a peak at 18:00, when the average daily temperature was 23.1 °C and the mean humidity was 52.5%. Even though many authors highlighted temperature as more detrimental in the diurnal fungal cycle, we believe that a minimum degree of humidity is still needed for release of each fungal species. Mean rainfall during the study period was the highest throughout the year, and as basidiomycetes spores are wet, their dispersal could be directly affected by precipitation. Sufficient moisture, either during rain periods or when humidity is high, may be required for the spore production of *Ganoderma* [71,74]. By the splash and ‘‘tap-and-puff’’ mechanism, raindrops hit the leaves and cause the attached spores to be released from their colonies into the air [74]. From this study, the sporulation of *Ganoderma* is more associated to increased moisture than humidity.

*Aspergillus* had a major peak pattern at 18:00. There is no significant diurnal periodicity of *Aspergillus/Penicillium* due to the nature of their spores, as they are dry, relatively small and thus, they can be released passively by minor wind speed [7]. Hameed et al. observed a double peaks pattern of *Aspergillus* at 10:00 and at 20:00 [16]. The highest concentration of *Aspergillus/Penicillium* occurred at 00:00 [74]. In the current study, *Ulocladium botrytis* peaked at midnight (00:00). Indeed, Al-Suwaine et al. also found that the concentration of *Ulocladium* is negatively correlated to temperature [78].

### 4.4. The Impact of Atmospheric Status (CO_2_ Concentration) on the Dynamics of Airborne Fungi

The concentrations of CO_2_ and other air pollutants increase with increased anthropogenic activities [79,80]. There were no significant differences in the composition and diversity of the airborne fungal population between the two study sites, though daily concentration of CO_2_ was significantly higher (*p* ≤ 0.001, *n* = 22) at the Industrial area site than at Qatar University Campus. In agreement with our results, in Taiwan, Chung et al. [79], and from the USA, Klamer et al., demonstrated that the fungal abundance and their species richness were not significantly influenced by elevated CO_2_ levels [81].

Although the main constituents of the airspora in the two study sites were attributed to similar fungal taxa, *Cladosporium, Ganoderma, Fusarium* and *Alternaria*, their deposition densities and distribution rate in the atmosphere are significantly different. *Cladosporium* showed higher and significant deposition density at the Qatar University site in comparison to *Fusarium* and *Alternaria*, which were highly abundant in the Industrial area site; however, *Ganoderma* had a similar relative abundance rate. This might be explained by the fact that elevated concentration of atmospheric CO_2_ raises the sporulation rate in some fungal genera [82], and might decline others, via changing fungal metabolism, and could subsequently inhibit their growth pattern, spore production and other reproduction processes [82]. Rather than the atmospheric CO_2_, other air pollutants, such as ozone (O_3_), nitrogen dioxide (NO_2_), sulphur dioxide (SO_2_) and particulate matter (PM_10_), might influence the availability and biological activities of airborne fungal taxa [80,83]. Indeed, the colony counts of *Alternaria* spp. and *Fusarium* spp. were significantly higher at the Industrial area site, which corresponds to a high CO_2_ level. Further agreement to our findings were the results of a study by Klironomos et al., suggesting that spore production levels of *Fusarium* spp. and *Alternaria* spp. were stimulated under elevated CO_2_ concentration levels [24]. Additionally, Wolf et al., in 2010, demonstrated that sporulation in *Alternaria alternata* increased at elevated CO_2_ concentration [25]. Indeed, increased CO_2_ concentration provides plants with a higher carbon/nitrogen ratio; in this way, pathogenic plant fungi accelerate their growth and sporulation rate [84]. Our results were based on 22 sampling exposures from each of the two study sites, which might not be enough to explicitly see the effects of CO_2_ and industrialization on dynamics of airspora, though a full year of monitoring is suggested for future research.

### 4.5. Respiratory Diseases and Airborne Fungal Spores

In the present study, *Cladosporium*, *Aspergillus*, *Fusarium*, *Alternaria*, *Ganoderma* spp. and *Penicillium* represented the major fungal airspora in the atmosphere of Doha, Qatar. Those fungi are frequently reported to have an association with allergy [40,65]. In the Middle East, and particularly in the Gulf region, only a few studies have attempted to assess the prevalence of allergic rhinitis, conjunctivitis, bronchial asthma and allergic bronchopulmonary mycoses developed from the frequent exposure to allergenic spores. In Qatar, Taj-Aldeen et al. investigated the allergic fungal rhinosinusitis (AFS) cases caused by *Aspergillus flavus*, where they found a huge quantity of allergic fungal mucin and dark crusts fully colonizing the sinuses, which required a course of systemic and topical corticosteroids [85]. In another Qatari study, the size of fungal infections at the public level have been estimated [86]. The data analysis revealed that 1486 people were affected by severe asthma with fungal sensitization, 1126 patients were diagnosed with allergic bronchopulmonary and 176 individuals complained of chronic pulmonary aspergillosis [86]. In Saudi Arabia, Hasnain et al. found relations between the high prevalence of asthma in children and the highest concentration of *Ganoderma* spp., *Cladosporium, Alternaria* and *Aspergillus* were believed to significantly contribute to causing allergic rhinitis and allergic asthma [56].

In the present study, the deposition density of *A. alternata* contributed to more than half of the total *Alternaria* spp. *A. alternata* is considered as one of the most significant aeroallergens [87]. Species of *A. fumigatus, A. niger, A. flavus* and *A.oryzae* were frequently correlated with the respiratory allergic cases, and particularly *A. flavus* due to its large spores, use to deposit in the upper respiratory tract and commonly cause fungal sinusitis [88]. *Cladosporium herbarum* is an important and main cause of inhalant fungal allergens, among other *Cladosporium* species [73]. In the present study, *C. herbarum* showed a minor deposition compared with other *Cladosporium* species.

## 5. Conclusions

In conclusion, our study demonstrated a seasonal pattern of airborne fungi in the atmosphere of Doha, Qatar. The main fungal airspora were attributed to *Cladosporium*, *Aspergillus*, *Fusarium* and *Alternaria*. Among the meteorological parameters, temperature might be the main determinant of the fungal spore incidence and diversity in the atmosphere of Doha. *Ganoderma* spp. is an allergic fungus which was reported for the first time and found to be a major constituent of Doha airspora during February and March. The study also revealed the intra-diurnal pattern of the airborne fungi in the atmosphere of Doha, Qatar, which indicates significant differences in the total colony counts and fungal diversity as a function of time. Both temperature and relative humidity affect the deposition densities of airspora. While other unmonitored air pollutants may have played various roles, here, we demonstrated that increased concentrations of atmospheric CO_2_ influenced the occurrence of certain fungal taxa. Considering the potentials effects of other air pollutants’ influence, it will be of interest to investigate their specific roles in the occurrence, dynamics and diversity of fungal taxa in Doha’s atmosphere. The present study is useful for filling the gap of data shortage on the dynamics of fungal taxa in Doha’s atmosphere, as well as providing baseline information for allergists, plant pathologists, meteorologists and other scientists. However, two major limitations can be deduced and should be considered for future investigations. The first is the fact that airspora data based on day-to-day spore trapping are more precise and complementary to the gravimetric method used in this study. The second limitation is that a two-month study is not enough to investigate the influence of higher CO_2_ concentration in the atmosphere on dynamics of airspora, as well as considering the influence of other important air-pollutants is essential.

## Figures and Tables

**Table 1:** Total colony count and occurrence of airborne fungi recovered from the air of Doha during the study period (1 April 2015 to 31 March 2016) using the gravimetric method (a total of 176 exposure samplings). Bold fonts refer to the genus and its related data.

Fungal Taxa	No. of Colonies	^(a)^ Colony Count (%)	^(b)^ Monthly Occurrence	Month of Presence
***Acremonium* sp.**	**3**	**0.25**	**1**	**December**
***Acrophialophora***	**4**	**0.33**	**1**	**June**
*Acrophialophora fusispora* (S.B. Saksena) Samson	4	0.33	1	June
***Agaricales* sp.**	**2**	**0.17**	**1**	**March**
***Alternaria***	**102**	**8.52**	**11**	**Yearly, except June**
*Alternaria alternata* (FR.) Keissler	62	5.18	11	Yearly, except June
*Alternaria brassicicola* (Schwein.) Wiltshire	3	0.25	1	February
*Alternaria chlamydospora* Mouchacca	13	1.09	3	December, January, February
*Alternaria infectoria* E.G. Simmons	12	1.00	1	March
*Alternaria phragmospora* V. Emden	2	0.17	2	February, April
*Alternaria porri* (Ellis) Cif.	4	0.33	1	December
*Alternaria tenuissima* (Kunze) Wilt.	6	0.50	2	May, July
***Aspergillus***	**124**	**10.36**	**12**	**Yearly**
*Aspergillus flavus* species complex	59	4.93	11	Yearly, except January
*Aspergillus fumigatus* species complex	2	0.17	1	November
*Aspergillus alutaceus* species complex	5	0.42	2	December, March
*Aspergillus terreus* species complex	3	0.25	2	April, May
*Aspergillus versicolor* species complex	35	2.92	5	
*Aspergillus niger* species complex	6	0.5	4	September, November, January, February
*Aspirgillus ustus* species complex	5	0.42	2	July, February
***Blastomyces* sp.**	**2**	**0.17**	**1**	**December**
***Cladosporium***	**721**	**60.23**	**12**	**Yearly**
*Cladosporium cladosporioides* species complex	509	42.52	12	Yearly
*Cladosporium herbarum* (Pers.) Link	4	0.33	3	October, December, February
*Cladosporium macrocarpum* Preuss	66	5.51	9	Yearly, except June, August and September
*Cladosporium oxysporum* Berk. & M.A. Curtis	13	1.09	4	February, March, April, May
*Cladosporium* sp.	80	6.68	1	April
*Cladosporium sphaerosprmum* Penzig	46	3.84	11	Yearly, except November
*Cladosporium tenuissimum* Cooke	3	0.25	3	November, February, March
***Cochliobolus***	**18**	**1.50**	**8**	**April, June, July, August, September, October, December**
*Cochliobolus australiencsis* (Tsuda & Ueyama) Alcorn	6	0.50	4	June, July, September
*Cochliobolus hawaiinsis* Alcorn (anamorph)	3	0.25	1	August
*Cochliobolus lunatus* Nelson & Haasis (anamorph)	4	0.33	1	April
*Cochliobolus spicifer* Nelson (anamorph)	5	0.42	2	October, December
***Epicoccum***	**2**	**0.17**	**2**	**May, June**
*Epicoccum nigrum*	2	0.17	2	May, June
***Fusarium***	**113**	**9.44**	**12**	**Yearly**
*Fusarium* sp.	76	6.35	8	January, February, March, April, May, October, November
*Fusarium chlamydosporum* Wollenweber & Reinking	10	0.84	5	November, December, January, February, March
*Fusarium dimerum* Penzig	8	0.67	5	April, May, December, February, March
*Fusarium moniliforme* (A. Braun) Wollenweber	3	0.25	1	October
*Fusarium oxysporum* species complex	16	1.34	6	October, November, December, February, March, April
***Ganoderma* spp.**	**28**	**2.34**	**2**	**February, March**
***Geotrichum***	**2**	**0.17**	**1**	**June**
*Geotrichum candidum* Link	2	0.17	1	June
***Mucor* sp.**	**2**	**0.17**	**1**	**March**
***Myrothecium***	**2**	**0.17**	1	December
*Myrothecium verrucaria* (Alb. & Schwein) Ditmar	2	0.17	1	December
***Penicillium***	**24**	**2.01**	**4**	**January, February, March, May, June**
***Phoma***	**4**	**0.33**	**1**	**March**
*Phoma glomerata* (Corda) Wollenw.	4	0.33	1	March
***Pleospora***	**7**	**0.58**	**3**	**April, May, December**
***Rhizopus***	**5**	**0.42**	**2**	**January, March**
*Rhizopus oryzae* Went & Prinsen-Geerligs	2	0.17	1	January
*Rhizopus stolonifer* (Ehrenb.) Lind	3	0.25	2	March
***Stachybotrys***	**6**	**0.50**	**2**	**December, March**
*Stachybotrys chartarum* (Ehrenb.) Hughes	3	0.25	1	December
*Stachybotrys elegans* (Pidopl.) W. Gams	3	0.25	1	March
***Thanatephorus***	**2**	**0.17**	**1**	**January**
*Thanatephorus cucumeris* (Frank) Donk	2	0.17	1	January
***Ulocladium***	**15**	**1.25**	**4**	
*Ulocladium botrytis* Preuss	9	0.75	2	March, July
*Ulocladium chartarum* (Preuss) Simmons	6	0.50	2	March, May
UNKNOWN	9	0.75	3	January, March, May
**MOULDS**	**1197**	**80.88**	12	Entire year
**YEASTS**	**283**	**19.12**	12	Entire year
**Total No. of Fungal Colonies**	**1480**			

^(a)^ Calculated as a percentage of the total count of mould colonies (1197) recovered from the entire study. ^(b)^ Number of months of occurrence out of 12 months.

**Table 2:** Monthly climatic parameters of Doha during the year of study versus results of correlation analysis. The correlation was accomplished based on daily data as well as monthly data and using Pearson correlation coefficient.

Month	Temperature (°C)			Mean Relative Humidity (%)	Total Rainfall (mm)	Mean Wind Speed (m/s)	Mean Wind Direction (Degree)
	Maximum	Minimum	Mean				
April 2015	35.5	23.6	29.0	27.6	0.0	4.3	255.7 = WSW ^a^
May	41.0	28.7	34.4	26.0	0.0	4.2	243.3 = WSW
June	42.2	29.8	35.8	31.7	0.0	4.3	297.1 = WNW
July	40.7	32.2	36.2	45.0	0.0	3.7	230.0 = SW
August	41.8	32.1	36.1	58.9	0.0	2.7	214.3 = SW
September	39.9	30.0	34.3	55.8	0.0	2.5	265.0 = W
October	36.7	27.2	31.4	59.9	0.0	2.7	308.3 = NW
November	29.4	21.3	25.0	66.7	0.0	2.4	317.8 = NW
December	23.7	15.9	19.5	69.7	0.2	3.0	292.5 = WNW
January 2016	22.9	14.3	18.1	66.3	0.1	3.1	256.3 = WSW
February	24.0	15.5	19.4	59.5	0.0	2.9	233.1 = SW
March	27.6	19.0	23.1	58.3	1.3	3.1	185.7 = S
**Correlation Coefficients/Daily Weather Data versus Daily Data of Number of Fungal Colonies**							
*Cladosporium*	−0.231 *	−0.228 *	−0.237 *	−0.0828	−0.0328	0.427 **	0.175
*Alternaria*	−0.243 *	−0.193 *	−0.222 *	0.0125	−0.0243	0.323 **	−0.111
*Aspergillus*	0.0135	0.0347	0.0209	0.0459	−0.0737	0.0779	0.0909
*Fusarium*	0.025	0.0906	0.0673	−0.0697	−0.0276	0.329 **	0.084
Total daily colony count	−0.23 *	−0.215 *	−0.231 *	−0.102	−0.0519	0.484 **	0.155
Total daily number of species	−0.26 **	−0.26 **	−0.267 **	−0.0249	−0.0917	0.257 **	0.143
**Correlation Coefficients/Monthly Weather Data versus Monthly Data of Total Number of Colonies**							
*Cladosporium*	−0.625 *	−0.703 *	−0.666 *	0.14	0.108	0.129	0.267
*Alternaria*	−0.203 **	−0.154 *	−0.18 **	0.36	0.562 **	−0.253	0.569
*Aspergillus*	−0.724	−0.696	−0.712	0.468	0.77	−0.141	−0.179
*Fusarium*	0.0846	0.0595	0.0821	−0.217	0.125	0.37	−0.348
Total monthly colony count	−0.684	0.189	0.189	0.189	0.307	0.115	0.181
Total monthly number of species	−0.642	−0.681	−0.66	0.173	0.468	0.022	−0.241

(**) Highly significant correlation at *p* ≤ 0.01, (*) Significant correlation at *p* ≤ 0.05. ^a^ W = West, E = East, N = North, S = South

**Table 3:** Diurnal variation of species richness and abundance (mean total colony count) of airborne fungi in the atmosphere of Doha, Qatar, with respect to variations in temperature and relative humidity. A total of 72 exposure samplings were obtained during the period 1 February to 31 March 2016.

Sampling Time	Mean Temp °C	Mean RH %	Total Colony Count *	Total Number of Fungal Species *
06:00	18.2	70.3	143 ^bc^	23 ^ab^
12:00	24.5	43	195 ^ab^	23 ^ab^
18:00	23.1	52.5	214 ^a^	30 ^a^
00:00	19.5	63.5	79 ^c^	15 ^b^

* Within each column, means followed by common letters are not significantly different at *p* = 0.05 according to Tukey’s test.

**Table 4:** Similarity * table among different diurnal times with respect to fungal species encountered from 72 exposure samplings in the atmosphere of Doha, Qatar, during the period 1 February to 31 March 2016.

Time	00:00	06:00	12:00	18:00
00:00	100%	65%	58%	41%
06:00	65%	100%	48%	56%
12:00	58%	48%	100%	39%
18:00	41%	56%	39%	100%

* Jaccard similarity coefficient = (c/(a + b) − c) × 100. c = number of common species between any two time periods (a and b).
